# Acute ingestion of a novel whey-derived peptide improves vascular endothelial responses in healthy individuals: a randomized, placebo controlled trial

**DOI:** 10.1186/1475-2891-8-34

**Published:** 2009-07-22

**Authors:** Kevin D Ballard, Richard S Bruno, Richard L Seip, Erin E Quann, Brittanie M Volk, Daniel J Freidenreich, Diana M Kawiecki, Brian R Kupchak, Min-Yu Chung, William J Kraemer, Jeff S Volek

**Affiliations:** 1 Department of Kinesiology, University of Connecticut, 2095 Hillside Road, Unit 1110, Storrs, CT, 06269, USA; 2 Department of Nutritional Sciences, University of Connecticut, Roy E. Jones Building, Unit 4017, Storrs, CT, 06269, USA

## Abstract

**Background:**

Whey protein is a potential source of bioactive peptides. Based on findings from *in vitro *experiments indicating a novel whey derived peptide (NOP-47) increased endothelial nitric oxide synthesis, we tested its effects on vascular function in humans.

**Methods:**

A randomized, placebo-controlled, crossover study design was used. Healthy men (n = 10) and women (n = 10) (25 ± 5 y, BMI = 24.3 ± 2.3 kg/m^2^) participated in two vascular testing days each preceded by 2 wk of supplementation with a single dose of 5 g/day of a novel whey-derived peptide (NOP-47) or placebo. There was a 2 wk washout period between trials. After 2 wk of supplementation, vascular function in the forearm and circulating oxidative stress and inflammatory related biomarkers were measured serially for 2 h after ingestion of 5 g of NOP-47 or placebo. Macrovascular and microvascular function were assessed using brachial artery flow mediated dilation (FMD) and venous occlusion strain gauge plethysmography.

**Results:**

Baseline peak FMD was not different for Placebo (7.7%) and NOP-47 (7.8%). Placebo had no effect on FMD at 30, 60, and 90 min post-ingestion (7.5%, 7.2%, and 7.6%, respectively) whereas NOP-47 significantly improved FMD responses at these respective postprandial time points compared to baseline (8.9%, 9.9%, and 9.0%; *P *< 0.0001 for time × trial interaction). Baseline reactive hyperemia forearm blood flow was not different for placebo (27.2 ± 7.2%/min) and NOP-47 (27.3 ± 7.6%/min). Hyperemia blood flow measured 120 min post-ingestion (27.2 ± 7.8%/min) was unaffected by placebo whereas NOP-47 significantly increased hyperemia compared to baseline (29.9 ± 7.8%/min; *P *= 0.008 for time × trial interaction). Plasma myeloperoxidase was increased transiently by both NOP-47 and placebo, but there were no changes in markers inflammation. Plasma total nitrites/nitrates significantly decreased over the 2 hr post-ingestion period and were lower at 120 min after placebo (-25%) compared to NOP-47 (-18%).

**Conclusion:**

These findings indicate that supplementation with a novel whey-derived peptide in healthy individuals improves vascular function.

## Background

The vascular endothelium is a single cell layer lining the lumen of blood vessels that substantially impacts vascular health and disease risk by regulating vasoconstriction and vasodilation, blood pressure, blood clotting, angiogenesis, inflammation, and passage of materials between the circulating blood and the interior components of the vessel wall. An important paracrine factor involved in vascular homoeostasis is endothelium-derived nitric oxide (NO·), which is a potent vasodilator that also inhibits platelet aggregation, inflammatory cell adhesion to the vessel wall, and smooth muscle cell proliferation [1]. Impaired NO· signaling and endothelial dysfunction have been implicated in metabolic syndrome [2] and cardiovascular disease [3]. Therefore, interventions that target NO· and vascular function are relevant for disease prevention.

Numerous therapies targeting the vascular endothelium have been proposed [4]. Nutritional strategies have focused on antioxidants (e.g., vitamin E, vitamin C, polyphenols, etc.) because oxidative stress contributes to endothelial dysfunction. Antioxidant supplementation mitigates postprandial endothelial dysfunction associated with high carbohydrate and high fat meals [5]. Oral supplementation [6] and intra-arterial administration [7] of L-arginine, the rate limiting amino acid for endothelial NO· synthesis [8], restored impaired endothelial function in older individuals and in response to a high fat meal [9], but not in younger adults with normal endothelial function [10].

Bioactive peptides derived from food, especially milk proteins, have been shown to exert a wide range of biological actions including decreased blood pressure [11,12] and improved endothelial function[13]. Milk is a rich source of angiotensin-converting enzyme (ACE) inhibitory peptides [14]. ACE inhibition prevents the conversion of angiotensin I to angiotensin II, a potent vasoconstrictor. Several clinical studies have shown improvement in endothelial function in patients prescribed ACE inhibitors [4], which could be the result of pleiotropic effects of ACE inhibitors on the vascular endothelium [15].

A search for isolates from whey protein hydrolysates that could increase NO production was carried out by Glanbia Nutritionals. A nitric oxide peptide (NOP-47) was identified that was shown to increase NO synthesis in vitro as determined by the analysis of NO· metabolites in human pulmonary artery endothelial cells (HPAE-26) (data provided by Glanbia Nutritionals). To extend upon these findings, we conducted a randomized, double-blind, cross-over trial to determine if NOP-47 affected vascular physiology in healthy human volunteers. We hypothesized that a single dose of NOP-47 would enhance vascular function as measured by flow-mediated dilation (FMD) of the brachial artery using high-frequency ultrasound [16] and reactive hyperemia forearm blood flow assessed by venous occlusion plethysmography [17]. Forearm FMD measures dilation in a conduit artery and is considered an index of NO· bioavailability [18] that is also correlated with coronary artery endothelial function [19] and cardiovascular disease risk and mortality [20]. Reactive hyperemia venous occlusion plethysmography measures vasodilation in the resistance vessels, and is not appreciably affected by NO· in human forearms [21]. A secondary objective was to characterize the effects of NOP-47 on circulating markers of antioxidant capacity, oxidative stress, and inflammation since these factors have been demonstrated to influence vascular function though various biologic mechanisms.

## Methods

### Study design

**Figure 1:**
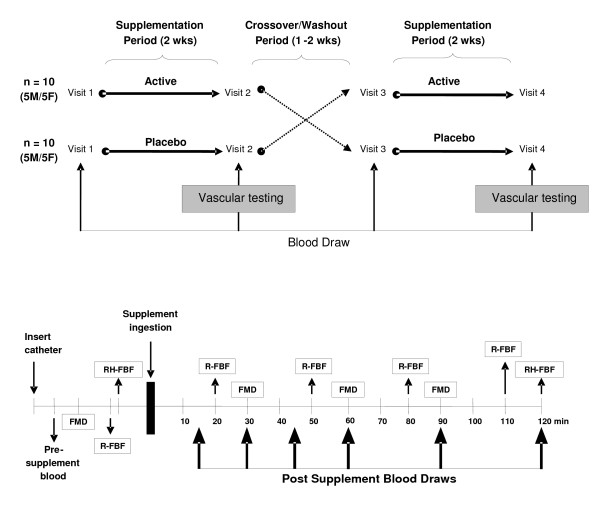
**Study timeline (A) and vascular testing protocol (B)**. FMD = flow mediated dilation; R-FBF = resting forearm blood flow; RH-FBF = reactive hyperemia forearm blood flow.

This study was approved by the Institutional Review Board for use of human subjects in research at the University of Connecticut. All subjects provided written informed consent after having the risks of the study carefully explained to them. A randomized, placebo-controlled, crossover study design with a washout period was conducted. Subjects participated in two vascular testing days with each preceded by 2 wk of daily supplementation with either a whey-derived peptide (NOP-47) or placebo. The order of supplementation was balanced. Following the completion of the first 2 wk supplementation period and first day of vascular testing, participants underwent a minimum 1 wk washout period after which they started the second 2 wk supplementation period consuming the alternative supplement. Each subject reported to the lab for four separate visits (Figure 1, **top**). In order to eliminate confounding influences on the experimental variables subjects were instructed to fast for 12 h, avoid alcohol, caffeine, and exercise for 24 h, and to consume 1 L of water the night before the visit and 480 mL the morning of the visit to ensure adequately hydration.

### Subjects

Healthy volunteers (*n *= 20) between 21–39 y were studied (Table 1). Exclusion criteria for subjects included overt chronic diseases as determined by medical history questionnaire, hypertension, smoking, use of vasoactive medications or supplements, or weight change greater than 2.3 kg in the past 3 mo. Women were screened to determine menstrual history and were excluded if hormonal contraceptive use was initiated or changed within the past 3 mo.

**Table 1:** Subject characteristics.

Age, yr	24.8 ± 4.5
Sex (M/F)	10/10
Height, cm	169.5 ± 9.4
Weight, kg	69.8 ± 9.1
BMI, kg·m^2^	24.3 ± 2.3
SBP, mmHg	110 ± 6
DBP, mmHg	68 ± 5
HR, bpm	57 ± 9
Waist Circumference, cm	77.8 ± 5.6

Values are means ± SD. BMI, body mass index; SBP, systolic blood pressure; DBP, diastolic blood pressure; HR, heart rate.

### Supplementation protocol

The active supplement was a proprietary peptide isolated from a whey protein hydrolysate (NOP-47, Glanbia Nutritionals, Twin Falls, ID) (Table 2). A daily dose of 5 g was pre-measured and placed in individual packets with artificial sweetener. The placebo was identical except the packets contained only artificial sweetener (aspartame and acesulfame potassium). Subjects were provided a 2 wk supply and instructed to consume one packet per day mixed in 300 mL water. Compliance was 100% as assessed by written documentation in log books and verified by study personnel. On the morning of vascular testing, subjects consumed one packet containing 5 g of NOP-47 or placebo mixed in water in the presence of an investigator not directly involved in vascular data collection nor analysis at the completion of the study. Subjects ingested the beverage within 3 min, after which a timer was started for the 2 h postprandial testing protocol. A questionnaire to address subjective symptoms and side effects associated with each supplement was administered at the end of the study.

**Table 2:** Amino acid composition of the whey peptide (NOP-47).

**Variables**	**NOP-47**
Tryptophan	1.33
Cystine	0.52
Methionine	5.05
Aspartic acid	5.46
Threonine	10.74
Serine	4.29
Glutamic acid	8.21
Proline	2.30
Glycine	1.18
Alanine	6.86
Valine	7.31
Isoleucine	5.69
Leucine	22.40
Tyrosine	2.31
Phenylalanine	4.41
Lysine	5.79
Histidine	1.70
Arginine	1.35

Values are g/100 g powder.

Five grams of powder was mixed with artificial sweetener in 300 mL water.

### Testing protocol

Upon arrival to the laboratory, participants provided a urine sample and hydration state was confirmed by measurement of urine specific gravity (USG) with a handheld refractometer. A USG < 1.020 indicated euhydration. If USG was > 1.020, then participants were instructed to drink water until their USG was < 1.020. Body mass was measured to the nearest 0.1 kg on a calibrated digital scale.

Visits 1 and 3 consisted of anthropometric measurements, detailed instructions on filling out dietary records, distribution of supplements, and a single venous blood draw obtained in a supine position. Visits 2 and 4 occurred on two occasions at the same time of the day after 2 wk supplementation with NOP-47 or placebo. A flexible catheter was inserted into a left forearm vein and after a 15 min supine stabilization period, blood samples were collected from a 3-way stopcock connected to the end of the catheter for fasting baseline and subsequent postprandial biochemistry measurements. Next fasting measurements of FMD and forearm blood flow (FBF) (described below) were determined. Fifteen minutes of recovery were allowed between FMD and FBF measurements before ingestion of the test beverage. Following these baseline measurements, subjects consumed a single 5 g dose of either NOP-47 or placebo mixed in 300 mL of water and with artificial sweetener. Post-ingestion FMD and FBF measurements were made intermittently (Figure 1, **bottom**). Blood samples were obtained at 15, 30, 45, 60, 90, and 120 min post-ingestion. Subjects remained supine in a comfortable position for the entire duration of the test. To ensure standardization between testing trials subjects were instructed to maintain their current level of physical activity during the study period and to replicate their dietary intake from previously recorded diet records the day prior to each vascular testing visit. Women were assessed during the same phase of their individual menstrual cycle as to account for any changes in vascular function [22] or blood markers due to menstrual phase.

### Flow mediated dilation

FMD was assessed using standardized procedures for performing high-frequency ultrasonographic imaging before (PRE) and at 30, 60, and 90 min after ingestion of the test beverage. The technique provokes the release of NO·, resulting in vasodilation that can be quantitated as an index of vasomotor function [16]. All tests were performed in a quiet, temperature-controlled room after a 10 min period in a supine position. A blood pressure cuff was placed on the upper right arm for occlusion. ECG leads were attached to monitor heart rate throughout the procedure. The brachial artery was imaged above the antecubital crease, and the transducer was placed to image the brachial artery in a longitudinal axis with clear visualization of the anterior and posterior vessel walls. When a clear image of the anterior and posterior walls of the artery was obtained, the transducer was held by a stereotactic clamp and the position held constant for the duration of the data collection. After optimization of the image, baseline brachial artery diameter was recorded for 30 heart beats. A mark was made on the arm where the image was collected. The cuff was inflated to 200 mm Hg for 5 min using a rapid cuff inflator (Hokanson E20, Bellevue, WA, USA) to occlude the brachial artery, and then released. Arterial diameter was then assessed continuously for 300 heart beats after occlusion[16]. Images of the brachial artery were obtained using an Acuson 13.0-MHz linear array transducer and an Aspen cardiac ultrasound system (Acuson Corp, Elmwood Park, NJ). Anatomical measurements were made to ensure placement of the transducer in the same location on the arm during the second visit. Image analysis was performed using MIA software (Medical Imaging Applications, Iowa City, IA, USA). For baseline, the average diameter taken from 30 frames was used. Three hundred frames were recorded from the postocclusion period. Peak postocclusion diameter was calculated by averaging the vessel diameter 5 frames immediately before the observed peak diameter and the 5 frames immediately after the same mark. Brachial artery FMD was calculated and expressed as a percentage of the baseline diameter [23]. All vascular measurements and analysis were performed by the same person. Using the same investigative team, coefficients of variation for arterial diameter on repeat scans with repositioning on a group of men and women (*N *= 10) in our laboratory were 2.2% for measurements made the same day, and 2.2% for measurements made on two consecutive days.

### Strain gauge plethysmography

Forearm blood flow was measured from the same arm as FMD using venous occlusion strain gauge plethysmography. A calibrated indium-gallium filled silastic strain gauge, encircled around the largest diameter of the right forearm, was connected to a plethysmograph (EC6, Hokanson, Inc., Bellevue, WA, USA). The increase in forearm volume was measured after blocking the venous efflux by an upper arm cuff inflated to 50 mmHg by a rapid cuff inflator (Hokanson E20, Bellevue, WA, USA) for 7 sec during each 15 sec cycle to determine resting forearm blood flow (R-FBF). This measurement was performed at rest (PRE) and in between the FMD protocol at 20, 50, 80, and 110 min after ingestion of the test beverage. The hand circulation was excluded by a wrist cuff inflated to 220 mmHg for 1 min before and during each flow evaluation. The forearm blood flow was estimated using specialized software (Noninvasive Vascular Program 3 (NIVP3), Hokanson, Bellevue, WA, USA) which calculated the slope from the change in forearm volume over time and determined blood flow as percent volume change per minute (%/min). Four plethysmographic measurements were averaged to obtain values for R-FBF. To determine reactive hyperemia induced forearm blood flow (RH-FBF), a blood pressure cuff on the upper right arm was inflated to a pressure of 200 mmHg for 5 min. FBF was determined as described above upon release of the occlusion. This measurement was performed at rest (PRE) and 120 min after ingestion of the test beverage.

### Blood collection and biochemical analyses

All blood samples were obtained from the left arm vein while participants rested quietly in the supine position. Whole blood was collected into tubes with no preservative or lithium heparin and centrifuged (1500 × g, 15 min, 4°C). Serum/plasma was transferred into storage tubes, snap frozen in liquid nitrogen, and stored at -80°C for future analysis. Samples for each asasy were analyzed in duplicate. Myeloperoxidase was measured from lithium heparin plasma by high-sensitivity sandwich ELISA (CardioMPO, Prognostix, Cleveland, OH) (CV = 5.9%). Serum glucose concentrations were analyzed using a YSI glucose/lactate analyzer (YSI 2300 STAT, Yellow Springs, OH). Total nitrite/nitrate (NO_2_^-^/NO_3_^-^) was measured as an estimate of NO· production using a colorimetric kit (Cayman Chemical, Ann Arbor, MI, USA) in accordance with the manufacturer's instructions (CV = 8.0%). Serum samples were filtered using 10 kDa molecular weight cut-off filters prior to analysis to reduce background absorbance. Plasma C-reactive protein (CRP) was determined on an IMMULITE Automated Analyzer using the commercially available immulite chemiluminescent enzyme immunometric assay (Immulite^®^, Diagnostic Products Corp., Los Angeles, CA, USA).

Total plasma antioxidant status was determined using the ferritin-reducing ability of plasma (FRAP) assay as previously described [24,25]. Briefly, diluted plasma (1:4) was mixed on a 96 well plate with 300 μl of freshly prepared and pre-warmed (37°C) FRAP reagent [50 ml of sodium acetate buffer (300 mmol/L), 5 ml of TPTZ reagent prepared in 40 mmol/L HCl, and 5 ml of FeCl_3 _(20 mmol/L)]. Following incubation (15 min, 37°C), samples were read at 593 nm on a microplate reader (SpectraMax M2, Molecular Devices Corporation, Sunnyvale, California, USA) and FRAP concentrations were calculated using trolox standards that were prepared in parallel (CV = 4.3%).

Plasma malondialdehyde (MDA) was measured by HPLC-FL as described previously [26] with minor modifications. In brief, 200 uL of plasma was mixed with 150 uL of 5% (w/v) TCA. After centrifugation (4,000 × g, 10 min, 4°C), the supernatant was thoroughly mixed with 50 μL of 0.6% (w/v) thiobarbituric acid. The sample was incubated (1 h, 100°C), then rapidly chilled in an ice bath, followed by the addition of 225 μL methanol and 25 μL 1 N NaOH. The supernatant was collected following centrifugation (16,000 × g, 4°C, 10 min) and subsequently injected (20 μl) on the HPLC system (Beckman Coulter; Fullerton, CA). The sample was separated on a C_18 _separation column (250 × 4.6 mm i.d.; 5 μm; Phenomenex; Torrance, CA) under isocratic (0.9 ml/min) conditions using 60:40 methanol and 50 mM phosphate buffer (pH 5.5) as the mobile phase. MDA was detected using excitation and emission settings of 532 nm and 553 nm, respectively, and was quantified against MDA standards that were prepared in parallel from 1,1,3,3-tetramethoxypropane.

Serum cytokines and chemokines [tumor necrosis factor-alpha (TNFα), interleukin-6 (IL-6), interleukin-8 (IL-8), monocyte chemoattractant protein-1 (MCP-1), vascular endothelial growth factor (VEGF), soluble E-selectin (sE-Selectin), soluble vascular cell adhesion molecule-1 (sVCAM-1), and soluble intracellular adhesion molecule-1 (sICAM-1)] were measured using xMAP^® ^technology on a Luminex^® ^IS 200 system with antibodies to these analytes from LINCO Research (St. Charles, MO)[27]. Assays were completed according to manufacturer's instructions.

### Statistical analyses

Forearm FMD data was analyzed with a 2 × 4 ANOVA with supplement trial (NOP-47 *vs *Placebo) and time (pre, 30, 60, and 90 min) as within effects. Sex was also included as an effect, but not found to be statistically significant and thus men and women were combined in all analyses. Significant main or interaction effects were further analyzed using a Fishers LSD *post hoc *test. Relationships among selected variables were examined using Pearson's product-moment correlation coefficient. The α-level for significance was set at 0.05.

## Results

Body mass remained stable over the course of the study (mean ± SD: 70.01 ± 9.27 kg versus 69.59 ± 8.85 kg for visits 1 and 4, respectively). There were also no significant changes in systolic or diastolic blood pressure after 2 wk of supplementation with NOP-47 and placebo. There were no adverse responses reported by subjects during either trial. All but one subject correctly identified which supplement contained NOP-47.

### Vascular function

**Figure 2:**
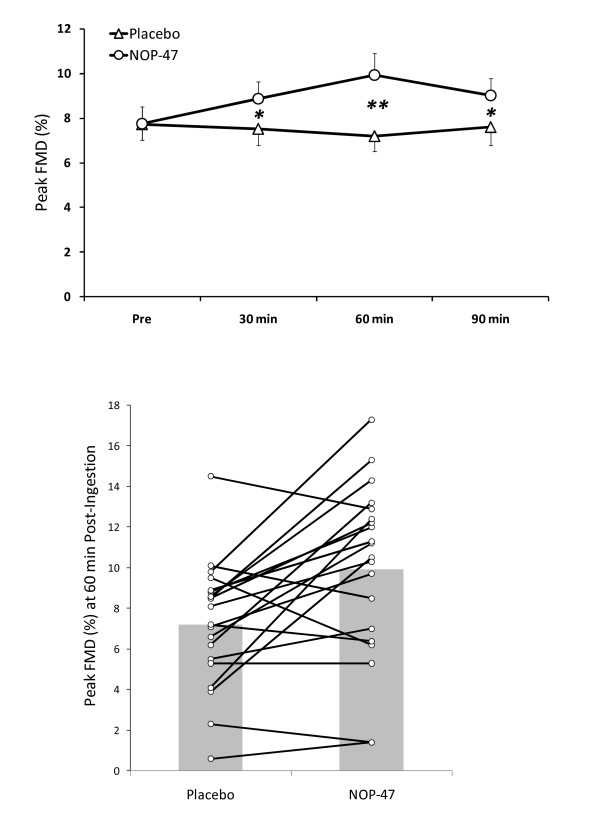
**FMD responses**. Mean (upper panel) and individual responses (lower panel) for peak flow mediated dilation (FMD) after ingestion of whey peptide (NOP-47) or Placebo. Significant differences between NOP-47 and Placebo (*P < 0.005, **P < 0.001).

Pre-occlusion diameters were not significantly different before ingestion of the NOP-47 (3.91 mm) and placebo (3.90 mm) and remained remarkably stable over time during both trials (range 3.89 to 3.91 mm) indicating maintenance in vascular tone over the postprandial period as well as a high degree of reproducibility in probe placement and measurement of the artery. Peak FMD (mean; 95% CI) significantly increased at 30 (8.87; 7.28–10.46%), 60 (9.94; 7.94–11.94%), and 90 (9.02; 7.41–10.63%) min post-ingestion in the NOP-47 trial which were significantly higher than corresponding placebo time points at 30 (7.52; 5.90–9.07%), 60 (7.21; 5.76–8.65%), and 90 (7.61; 5.91–9.31%) min (*P *< 0.0001 for time × trial interaction) (Figure 2, **top**). Individual responses revealed that 15 out of 20 subjects had greater peak FMD at 60 min (Figure 2, **bottom**) and 90 min post-NOP-47 ingestion compared to these same time points following ingestion of placebo.

**Figure 3:**
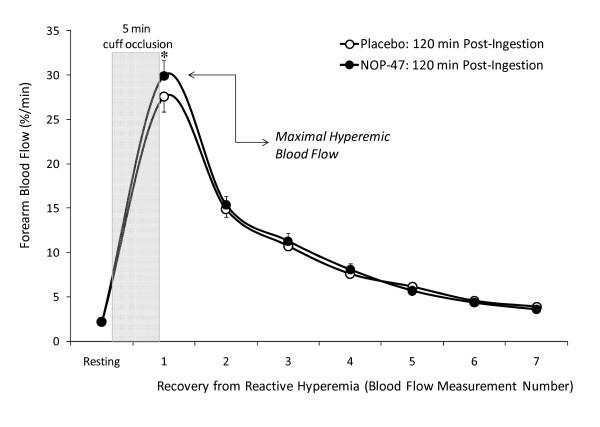
**Forearm blood flow responses**. Reactive hyperemia forearm blood flow was assessed using venous occlusion plethysmography 120 min after ingestion of whey peptide (NOP-47) Placebo. At each time point, reactive hyperemia induced forearm blood flow was assessed after 5 min of upper arm occlusion. Recovery of reactive hyperemic blood flow was determined after blocking the venous efflux of the upper arm for 7 sec during each of 8 subsequent 15-second cycles. Significant differences between NOP-47 and Placebo (*P < 0.05).

Reactive hyperemia forearm blood flow was assessed in response to 5 min of cuff occlusion by venous occlusion plethysmography before and 120 min after supplement ingestion. Maximal hyperemic blood flow was similar after 2 weeks of supplementation with NOP-47 (27.6 ± 7.6%/min) and placebo (27.6 ± 7.2%/min). The response to acute ingestion showed a significant increase at 120 min for NOP-47 (29.9 ± 7.5; 95% CI = 26.25–33.58%/min) and no change for placebo (27.5 ± 7.8; 95% CI = 23.51–30.84%/min) (*P *= 0.008 for time × trial interaction) (Figure 3). Resting forearm blood flow was also assessed before supplement ingestion and 20, 50, 80, and 110 min post-ingestion. Compared to hyperemic blood flow, resting blood flow values were considerably smaller in magnitude and only showed a significant main time effect (*P *= 0.002) as reflected by a significant increase at 110 min compared to pre-ingestion.

### Hematological responses

**Figure 4:**
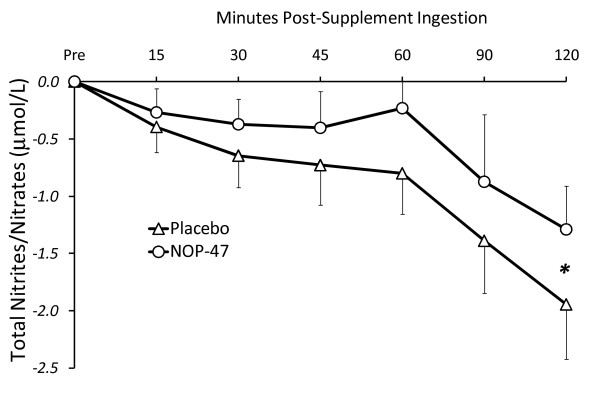
**Plasma total nitrites/nitrates (NOx), normalized to baseline, responses to ingestion of a whey peptide (NOP-47) or Placebo**. There was a significant main time effect. Significant differences between NOP-47 and Placebo (*P < 0.05).

Hematoligical responses are presented in Table 3. Serum glucose was unaffected by 2 wk of supplementation or acute ingestion. Plasma nitrites/nitrates (NO_x_) decreased significantly over time (*P *< 0.001; Figure 4) and specific post-hoc effects were observed at 90 and 120 min compared to pre-ingestion. There was a greater decline in subjects consuming the placebo at the 120 min time point (*P *= 0.03). There was a significant time effect for serum FRAP (*P *= 0.001) with values at 30 min higher than baseline. Fasting plasma MPO was not affected by 2 wk of supplementation. However, there was a significant main time effect in response to acute supplementation ingestion with values increasing significantly at 60 and 90 min (*P *= 0.003 for time effect). Plasma CRP, MDA and several inflammatory markers were unaffected by chronic or acute ingestion (Table 3).

**Table 3:** Glycemic, oxidative stress, and inflammatory responses to whey peptide (NOP-47) and placebo.

**Variables**	**NOP-47**	**Placebo**
Serum Glucose (mg/dL)		
Pre (Pre-Supplementation)	93.2 ± 6.5	96.6 ± 9.0
Pre-Ingestion (2 wk Post)	93.9 ± 7.6	93.6 ± 7.0
15 min	93.7 ± 7.6	92.8 ± 6.0
30 min	94.1 ± 7.11	92.5 ± 6.1
45 min	93.5 ± 6.7	92.3 ± 5.8
60 min	93.0 ± 6.5	92.6 ± 7.0
90 min	93.7 ± 6.4	92.7 ± 6.6
120 min	93.4 ± 6.9	91.2 ± 6.5
Serum FRAP (μmol/L of Trolox Equivalents)		
Pre (Pre-Supplementation)	364 ± 93	385 ± 78
Pre-Ingestion (2 wk Post)	354 ± 82	347 ± 67
15 min	370 ± 82	359 ± 76
30 min*	375 ± 80	369 ± 86
45 min	351 ± 78	366 ± 86
60 min	364 ± 76	358 ± 72
90 min	341 ± 73	359 ± 79
120 min	349 ± 76	349 ± 69
Plasma MPO (pmol/L)		
Pre (Pre-Supplementation)	397 ± 162	362 ± 116
Pre-Ingestion (2 wk Post)	374 ± 117	347 ± 123
15 min	372 ± 118	361 ± 136
30 min	382 ± 145	358 ± 126
45 min	400 ± 132	366 ± 124
60 min*	431 ± 136	390 ± 133
90 min*	420 ± 125	394 ± 112
120 min	392 ± 135	377 ± 115
Plasma CRP (mg/dL)		
Pre (Pre-Supplementation)	1.05 ± 0.88	1.75 ± 3.14
Pre-Ingestion (2 wk Post)	1.24 ± 1.51	1.11 ± 1.42
15 min	1.33 ± 1.73	1.01 ± 1.23
30 min	1.33 ± 1.77	1.14 ± 1.59
45 min	1.29 ± 1.75	1.15 ± 1.61
60 min	1.31 ± 1.77	1.08 ± 1.45
90 min	1.31 ± 1.75	1.09 ± 1.46
120 min	1.24 ± 1.65	1.02 ± 1.43
Plasma MDA (μmol/L)		
Pre (Pre-Supplementation)	0.31 ± 0.13	0.37 ± 0.16
Pre-Ingestion (2 wk Post)	0.32 ± 0.13	0.31 ± 0.13
15 min	0.31 ± 0.13	0.30 ± 0.12
30 min	0.33 ± 0.13	0.30 ± 0.11
45 min	0.34 ± 0.14	0.31 ± 0.12
60 min	0.32 ± 0.12	0.30 ± 0.14
90 min	0.32 ± 0.15	0.30 ± 0.15
120 min	0.32 ± 0.16	0.30 ± 0.14
sE-selectin (ng/mL)		
Pre (Pre-Supplementation)	22.6 ± 7.4	20.7 ± 8.5
Pre-Ingestion (2 wk Post)	19.6 ± 7.3	19.2 ± 7.3
60 min	20.0 ± 8.5	19.0 ± 7.6
120 min	19.7 ± 8.7	20.4 ± 8.6
sICAM-1 (ng/mL)		
Pre (Pre-Supplementation)	104 ± 17	95 ± 25
Pre-Ingestion (2 wk Post)	100 ± 21	97 ± 13
60 min	96 ± 16	94 ± 21
120 min	96 ± 16	97 ± 17
sVCAM-1 (ng/mL)		
Pre (Pre-Supplementation)	756 ± 145	732 ± 125
Pre-Ingestion (2 wk Post)	712 ± 124	709 ± 143
60 min	701 ± 112	700 ± 144
120 min	710 ± 118	719 ± 186
IL-6 (pg/mL)		
Pre (Pre-Supplementation)	493 ± 1162	473 ± 1077
Pre-Ingestion (2 wk Post)	417 ± 1002	492 ± 1238
60 min	563 ± 1441	504 ± 1266
120 min	579 ± 1536	483 ± 1133
IL-8 (pg/mL)		
Pre (Pre-Supplementation)	158 ± 243	139 ± 213
Pre-Ingestion (2 wk Post)	105 ± 183	159 ± 288
60 min	155 ± 323	164 ± 300
120 min	187 ± 370	183 ± 331
MCP-1 (pg/mL)		
Pre (Pre-Supplementation)	435 ± 306	305 ± 132
Pre-Ingestion (2 wk Post)	428 ± 282	427 ± 244
60 min	447 ± 322	423 ± 276
120 min	398 ± 286	427 ± 316
TNF-alpha (pg/mL)		
Pre (Pre-Supplementation)	39 ± 70	43 ± 89
Pre-Ingestion (2 wk Post)	19 ± 35	41 ± 105
60 min	39 ± 93	47 ± 120
120 min	63 ± 193	54 ± 141
VEGF (pg/mL)		
Pre (Pre-Supplementation)	1320 ± 1394	1189 ± 1328
Pre-Ingestion (2 wk Post)	994 ± 1080	1206 ± 1435
60 min	1096 ± 1278	1201 ± 1401
120 min	1206 ± 1406	1273 ± 1548

Values are mean ± SD. *Significant main time effect, *P *< 0.05 from Pre-Ingestion.

FRAP = ferric-reducing ability of plasma; MPO = myeloperoxidase; CRP = C-reactive protein; MDA = malondialdehyde; sICAM-1 = soluble intercellular adhesion molecule-1; sVAM-1 = soluble vascular adhesion molecule-1; IL-6 = interleukin-6; MCP-1 = monocyte chemotactic protein-1; TNF-alpha = tumor necrosis factor alpha; VEGF = Vascular endothelial growth factor.

## Discussion

We tested the effects of a novel peptide derived from whey on vascular endothelial function in healthy, young men and women. Peripheral vascular function was assessed in a conduit vessel using FMD of the brachial artery by high-resolution ultrasound and in forearm resistance vessels using venous occlusion plethysmography. We demonstrated that 2 wk of supplementation had no effect on fasting measures of vascular function, but acute ingestion of NOP-47 significantly increased postprandial FMD at 30, 60 and 90 min post-ingestion and reactive hyperemia forearm blood flow measured at 120 min post-ingestion. Shear stress induced dilation of conduit vessels like the brachial artery are principally regulated by the potent vasodilator NO· [28], whereas dilation of resistance vessels in response to reactive hyperemia is largely independent of NO· [21]. Therefore acute ingestion of NOP-47 likely enhanced vascular endothelial function through mechanisms that were dependent as well as independent of NO·.

To define the NOP-47 mediated activity on vasodilation, we measured NO· status by evaluating total nitrite and nitrate levels (NO_x_), the final metabolites of NO·. We detected a time-dependent decrease in plasma NO_x _during the 2 h testing period that was partly inhibited after ingestion of NOP-47 at 120 min (Figure 4). Previous studies have shown a decline in postprandial NO_x _and decreased FMD after both high fat and high carbohydrate meals[29]. Lower NOx levels correlate with reduced FMD in patients with endothelial dysfunction [30] and in healthy young men and women [31]. Whether better maintenance of NO· after NOP-47 ingestion contributed to the enhanced vascular responses in this study remains unclear. Although NO_x _correlates with FMD, the assay is not specific for endothelial NO· production and could also reflect NO· derived from neuronal and inducible NO· synthase [32]. Alternatively, other factors besides NO· may be responsible for the enhanced dilation. Alterations in the balance between vasodilators (e.g., bradykinin, adenosine, vascular endothelial growth factor, and prostacyclin) and vasoconstrictors (e.g., endothelin, prostanoids, and angiotensin II) have been suggested to contribute to the FMD response [33]. Experiments that involve infusion of N^G^-nitro-L-arginine methyl ester (L-NAME), an inhibitor of NO synthesis, would help to elucidate whether the enhanced FMD response to NOP-47 is NO·-dependent.

Whey derived peptides showing ACE inhibitory effects are released during normal digestion in the gastrointestinal tract by proteases. Commonly used enzymatic procedures in the manufacturing of whey hydrolysates also result in rich sources of ACE inhibitory peptides. In order for oral ingestion of whey peptides to exert hypotensive or other biological effects *in vivo*, it must be absorbed intact and be transported to the target tissue while escaping destruction from intestinal brush border or serum peptidases. Evidence exists to support that peptides are absorbed intact through the intestine by paracellular and transcellular routes[34]. Indeed, a specific whey-derived heptapeptide having ACE inhibitory activity was demonstrated to be bioavailable [35].

A possible mechanism by which whey peptides might improve endothelial function is through ACE inhibition. Human clinical trials have shown improvement in endothelial function in patients taking ACE inhibitors (reviewed in [4]), which could be the result of pleiotropic effects of ACE inhibitors on the vascular endothelium [15]. A further mechanism by which whey peptides could affect vascular function is by increasing arginine availability, the rate limiting substrate for nitric oxide synthesis. However, NOP-47 contained approximately 135 mg of arginine, well below the doses previously demonstrated to improve FMD [36].

Most nutraceutical interventions that reported favorably effects on FMD were studied after the ingestion of meals that induced oxidative and inflammatory stress to the endothelium leading to vascular dysfunction[5]. In this study, our primary objective was to examine the effects of NOP-47 in the fasted state without the confounding effects of other nutrients in healthy non-hypertensive individuals with presumed normal endothelial functioning. There was however a significant increase of ~15% in plasma MPO one hour after ingestion of both NOP-47 and placebo suggesting the protocol induced a small transient elevation in neutrophil activation and oxidative stress. The ischemia caused by repeated forearm arterial and venous occlusions performed during the vascular function tests may have produced brief periods of turbulent flow causing an increase in MPO[37,38]. The increase in MPO at 60 and 90 min after ingestion of both NOP-47 and placebo coincided in time with the significant decrease in NOx. Elevated MPO levels have been shown to interfere with endothelial NO· action and are highly associated with impaired FMD[39].

## Conclusion

The results of this preliminary study suggests that in individuals with normal endothelial function, the acute ingestion of a peptide derived from whey improves both conduit and resistance vascular responses. Ingestion of NOP-47 enhanced vascular function in the context of minimal changes in glucose and markers of oxidative stress and inflammation. The peptide could be of value in populations with vascular dysfunction or as a method to attenuate the vascular dysfunction associated with the postprandial period. Future experiments that explore the impact of NOP-47 on postprandial vascular function during hyperglycemia or hypertriglyceridemia with resulting oxidative and inflammatory stress or studies that specifically address the therapeutic potential in patients with vascular dysfunction would be informative.

## Competing interests

Glanbia Nutritionals provided funding for the study and supplied the test supplements used in the study.

## Authors' contributions

KDB contributed to study conception and design, acquisition of data, analysis and interpretation of data and drafting and revising the manuscript. RSB, RLS and WJK contributed to study conception and design and analysis and interpretation of data. EEQ, BMV, DJF, DMK, M-YC and BRK assisted with data acquisition and analysis. JSV contributed to study conception and design, interpretation of data and drafting and revising the manuscript. All authors read and approved the final manuscript.

## References

[B1] Cooke JP (2004). The pivotal role of nitric oxide for vascular health. Can J Cardiol.

[B2] Steinberg HO, Chaker H, Leaming R, Johnson A, Brechtel G, Baron AD (1996). Obesity/insulin resistance is associated with endothelial dysfunction. Implications for the syndrome of insulin resistance. J Clin Invest.

[B3] Le Brocq M, Leslie SJ, Milliken P, Megson IL (2008). Endothelial dysfunction: from molecular mechanisms to measurement, clinical implications, and therapeutic opportunities. Antioxid Redox Signal.

[B4] Tousoulis D, Antoniades C, Koumallos N, Marinou K, Stefanadi E, Latsios G, Stefanadis C (2006). Novel therapies targeting vascular endothelium. Endothelium.

[B5] Lee IK, Kim HS, Bae JH (2002). Endothelial dysfunction: its relationship with acute hyperglycaemia and hyperlipidemia. Int J Clin Pract Suppl.

[B6] Bode-Boger SM, Muke J, Surdacki A, Brabant G, Boger RH, Frolich JC (2003). Oral L-arginine improves endothelial function in healthy individuals older than 70 years. Vasc Med.

[B7] Chauhan A, More RS, Mullins PA, Taylor G, Petch C, Schofield PM (1996). Aging-associated endothelial dysfunction in humans is reversed by L-arginine. J Am Coll Cardiol.

[B8] Yamashita H, Takenoshita M, Sakurai M, Bruick RK, Henzel WJ, Shillinglaw W, Arnot D, Uyeda K (2001). A glucose-responsive transcription factor that regulates carbohydrate metabolism in the liver. Proc Natl Acad Sci USA.

[B9] Marchesi S, Lupattelli G, Siepi D, Roscini AR, Vaudo G, Sinzinger H, Mannarino E (2001). Oral L-arginine administration attenuates postprandial endothelial dysfunction in young healthy males. J Clin Pharm Ther.

[B10] Adams MR, Forsyth CJ, Jessup W, Robinson J, Celermajer DS (1995). Oral L-arginine inhibits platelet aggregation but does not enhance endothelium-dependent dilation in healthy young men. J Am Coll Cardiol.

[B11] Erdmann K, Cheung BW, Schroder H (2008). The possible roles of food-derived bioactive peptides in reducing the risk of cardiovascular disease. J Nutr Biochem.

[B12] FitzGerald RJ, Murray BA, Walsh DJ (2004). Hypotensive peptides from milk proteins. J Nutr.

[B13] Hirota T, Ohki K, Kawagishi R, Kajimoto Y, Mizuno S, Nakamura Y, Kitakaze M (2007). Casein hydrolysate containing the antihypertensive tripeptides Val-Pro-Pro and Ile-Pro-Pro improves vascular endothelial function independent of blood pressure-lowering effects: contribution of the inhibitory action of angiotensin-converting enzyme. Hypertens Res.

[B14] FitzGerald RJ, Meisel H (2000). Milk protein-derived peptide inhibitors of angiotensin-I-converting enzyme. Br J Nutr.

[B15] Faggiotto A, Paoletti R (1999). State-of-the-Art lecture. Statins and blockers of the renin-angiotensin system: vascular protection beyond their primary mode of action. Hypertension.

[B16] Corretti MC, Anderson TJ, Benjamin EJ, Celermajer D, Charbonneau F, Creager MA, Deanfield J, Drexler H, Gerhard-Herman M, Herrington D, Vallance P, Vita J, Vogel R (2002). Guidelines for the ultrasound assessment of endothelial-dependent flow-mediated vasodilation of the brachial artery: a report of the International Brachial Artery Reactivity Task Force. J Am Coll Cardiol.

[B17] Wilkinson IB, Webb DJ (2001). Venous occlusion plethysmography in cardiovascular research: methodology and clinical applications. Br J Clin Pharmacol.

[B18] Faulx MD, Wright AT, Hoit BD (2003). Detection of endothelial dysfunction with brachial artery ultrasound scanning. Am Heart J.

[B19] Anderson TJ, Uehata A, Gerhard MD, Meredith IT, Knab S, Delagrange D, Lieberman EH, Ganz P, Creager MA, Yeung AC (1995). Close relation of endothelial function in the human coronary and peripheral circulations. J Am Coll Cardiol.

[B20] Celermajer DS, Sorensen KE, Bull C, Robinson J, Deanfield JE (1994). Endothelium-dependent dilation in the systemic arteries of asymptomatic subjects relates to coronary risk factors and their interaction. J Am Coll Cardiol.

[B21] Tagawa T, Imaizumi T, Endo T, Shiramoto M, Harasawa Y, Takeshita A (1994). Role of nitric oxide in reactive hyperemia in human forearm vessels. Circulation.

[B22] Hashimoto M, Akishita M, Eto M, Ishikawa M, Kozaki K, Toba K, Sagara Y, Taketani Y, Orimo H, Ouchi Y (1995). Modulation of endothelium-dependent flow-mediated dilatation of the brachial artery by sex and menstrual cycle. Circulation.

[B23] Sonka M, Liang W, Lauer RM (2002). Automated analysis of brachial ultrasound image sequences: early detection of cardiovascular disease via surrogates of endothelial function. IEEE Trans Med Imaging.

[B24] Bruno RS, Ramakrishnan R, Montine TJ, Bray TM, Traber MG (2005). {alpha}-Tocopherol disappearance is faster in cigarette smokers and is inversely related to their ascorbic acid status. Am J Clin Nutr.

[B25] Benzie IF, Strain JJ (1996). The ferric reducing ability of plasma (FRAP) as a measure of "antioxidant power": the FRAP assay. Anal Biochem.

[B26] Young IS, Trimble ER (1991). Measurement of malondialdehyde in plasma by high performance liquid chromatography with fluorimetric detection. Ann Clin Biochem.

[B27] Liu MY, Xydakis AM, Hoogeveen RC, Jones PH, Smith EO, Nelson KW, Ballantyne CM (2005). Multiplexed analysis of biomarkers related to obesity and the metabolic syndrome in human plasma, using the Luminex-100 system. Clin Chem.

[B28] Joannides R, Haefeli WE, Linder L, Richard V, Bakkali EH, Thuillez C, Luscher TF (1995). Nitric oxide is responsible for flow-dependent dilatation of human peripheral conduit arteries in vivo. Circulation.

[B29] Blendea MC, Bard M, Sowers JR, Winer N (2005). High-fat meal impairs vascular compliance in a subgroup of young healthy subjects. Metabolism.

[B30] Kleinbongard P, Dejam A, Lauer T, Jax T, Kerber S, Gharini P, Balzer J, Zotz RB, Scharf RE, Willers R, Schechter AN, Feelisch M, Kelm M (2006). Plasma nitrite concentrations reflect the degree of endothelial dysfunction in humans. Free Radic Biol Med.

[B31] Casey DP, Beck DT, Braith RW (2007). Systemic plasma levels of nitrite/nitrate (NOx) reflect brachial flow-mediated dilation responses in young men and women. Clin Exp Pharmacol Physiol.

[B32] Ishibashi T, Yoshida J, Nishio M (1999). Evaluation of NOx in the cardiovascular system: relationship to NO-related compounds in vivo. Jpn J Pharmacol.

[B33] Deanfield JE, Halcox JP, Rabelink TJ (2007). Endothelial function and dysfunction: testing and clinical relevance. Circulation.

[B34] Vermeirssen V, Van Camp J, Verstraete W (2004). Bioavailability of angiotensin I converting enzyme inhibitory peptides. Br J Nutr.

[B35] Vermeirssen V, Deplancke B, Tappenden KA, Van Camp J, Gaskins HR, Verstraete W (2002). Intestinal transport of the lactokinin Ala-Leu-Pro-Met-His-Ile-Arg through a Caco-2 Bbe monolayer. J Pept Sci.

[B36] Bai Y, Sun L, Yang T, Sun K, Chen J, Hui R (2009). Increase in fasting vascular endothelial function after short-term oral L-arginine is effective when baseline flow-mediated dilation is low: a meta-analysis of randomized controlled trials. Am J Clin Nutr.

[B37] Rose S, Fiebrich M, Weber P, Dike J, Buhren V (1998). Neutrophil activation after skeletal muscle ischemia in humans. Shock.

[B38] De Keulenaer GW, Chappell DC, Ishizaka N, Nerem RM, Alexander RW, Griendling KK (1998). Oscillatory and steady laminar shear stress differentially affect human endothelial redox state: role of a superoxide-producing NADH oxidase. Circ Res.

[B39] Vita JA, Brennan ML, Gokce N, Mann SA, Goormastic M, Shishehbor MH, Penn MS, Keaney JF, Hazen SL (2004). Serum myeloperoxidase levels independently predict endothelial dysfunction in humans. Circulation.

